# A Longitudinal Study of the Predictors of Perceived Procedural Justice in Australian University Staff

**DOI:** 10.3389/fpsyg.2016.01271

**Published:** 2016-08-25

**Authors:** Silvia Pignata, Anthony H. Winefield, Chris Provis, Carolyn M. Boyd

**Affiliations:** ^1^School of Engineering, University of South AustraliaAdelaide, SA, Australia; ^2^Asia Pacific Centre of Work Health and Safety, School of Psychology, Social Work and Social Policy, University of South AustraliaAdelaide, SA, Australia; ^3^School of Psychology, University of AdelaideAdelaide, SA, Australia; ^4^School of Management, University of South AustraliaAdelaide, SA, Australia

**Keywords:** procedural justice, psychological contract, reciprocity, job satisfaction, organizational policy

## Abstract

**Purpose:** This study examined the factors that predict employees' perceptions of procedural justice in university settings. The paper also reviews the ethical aspects of justice and psychological contracts within employment relationships.

**Design/Methodology/Approach:** The study examined the predictors of perceived procedural justice in a two-wave longitudinal sample of 945 employees from 13 universities by applying the Job Demands-Resources theoretical model of stress. The proposed predictors were classified into two categories: Job demands of work pressure and work-home conflict; and job resources of job security, autonomy, trust in senior management, and trust in supervisor. The predictor model also examined job satisfaction and affective organizational commitment, demographic (age, gender, tenure, role) and individual characteristics (negative affectivity, job involvement) as well as Time 1 (T1) perceptions of procedural justice to ensure that tests were rigorous.

**Findings:** A series of hierarchical multiple regression analyses found that job satisfaction at T1 was the strongest predictor of perceived procedural justice at Time 2. Employees' trust in senior management, and their length of tenure also positively predicted justice perceptions. There were also differences between academic and non-academic staff groups, as non-academic employees' level of job satisfaction, trust in senior management, and their length of organizational tenure predicted procedural justice perceptions, whereas for academics, only job satisfaction predicted perceived justice. For the “all staff” category, job satisfaction was a dominant and enduring predictor of justice, and employees' trust in senior management also predicted justice.

**Research limitations/implications:** Results highlight the importance of workplace factors in enhancing fair procedures to encourage reciprocity from employees. As perceived procedural justice is also conceptually linked to the psychological contract between employees-employers, it is possible that employees' levels of job satisfaction and perceptions of trust in senior management, relative to other work attitudinal outcomes, may be more effective for improving the broader working environment, and promoting staff morale.

**Originality/value:** This study adds to research on applied business ethics as it focuses on the ethical aspects of perceived procedural justice and highlights the importance of workplace factors in enhancing fair procedures in organizational policy to encourage reciprocity and promote healthy organizational environments.

## Introduction

Recent international research shows that the high global costs of work-related stress for employers due to absenteeism, presenteeism, and the loss of productivity, and the subsequent high health care costs for the community are a growing international concern (Giorgi et al., 2014; Mucci et al., 2015). In addition, work-related stress can influence the development of physical health problems with consequent negative repercussions on the productivity of human resources (Mucci et al., 2014; Bjørnstad et al., 2015). On the other hand, research on healthy organizational environments demonstrates that employees who are psychologically attached to an organization work more effectively and contribute to achieving organizational goals through higher levels of performance (Somech and Drach-Zahavy, 2004). Indeed, organizations rely upon “…acts of cooperation, altruism, and spontaneous unrewarded help” from employees to maintain a healthy organizational environment (O'Reilly and Chatman, 1986, p. 493). Justice is an important theme in effective organizational contexts, and an improved knowledge of employees' experiences of justice is needed to help understand the benefits of developing and maintaining a sense of justice within organizations. Employees perceive three main forms of justice: The fairness of the outcomes of a decision or allocation of goods (distributive justice); the fairness of the processes that are used to make decisions (procedural justice); and the fairness of the interactions and treatment that an individual receives from another (relational justice; Li and Cropanzano, 2009).

### Organizational justice

The justice literature emphasizes the importance of increasing justice via fair procedures both for reasons of intrinsic ethicality, and to encourage reciprocity from employees and the subsequent benefits for organizations in promoting commitment to the organization and encouraging employee trust (Ambrose and Cropanzano, 2003; Holtz and Harold, 2009). We argue that studying justice and pro-organizational behaviors helps guide research to improve our understanding of these behaviors. This paper focuses on examining the predictors of employees' perceptions of the procedural aspect of organizational justice and contributes to the literature in two ways. First, it examines the factors (workplace and individual) that predict employee perceptions of procedural justice in the university context. The paper also reviews the ethical aspects of justice and psychological contracts within employment relationships in order to understand what types of factors individuals perceive to be fair and just. Thus, the aim of the present study is to bridge gaps in the literature as an improved knowledge of these characteristics may produce healthier work environments within complex organizations such as universities, as well as other organizations.

Procedural justice refers to how decisions affecting staff are made and whether the outcomes from those decisions are correct and fair (Greenberg, 1994). The rights and responsibilities of employees and employers exceed economic issues such as productivity and competitiveness, since employment relationships also require fair treatment, equity, and voice for employees (Budd, 2004). Employers' recognition of employee voice may be seen as an acceptance of their rights at work as their voice is expected to be able to influence decisions (Thibaut and Walker, 1975). Procedural justice perceptions assist in shaping employees' relationships with their employers (Folger and Cropanzano, 1998) and thus affect attitudes such as job satisfaction (Masterson et al., 2000), organizational commitment, trust in management (Folger and Konovsky, 1989), organizational citizenship behavior (OCB), and turnover intentions (Ambrose and Cropanzano, 2003). Committed employees are prepared to engage in OCB (Organ and Konovsky, 1989), which is discretionary behavior directed at individuals or toward the organization as a whole that goes beyond the formal expectations of the work role, and which benefits, or is intended to benefit, the organization (Organ, 1988). Meta-analyses suggest that employees are more likely to engage in OCB when they are fairly treated, have a good relationship with their supervisor and have high levels of affective commitment to their organization (Hoffman et al., 2007). Rhoades and Eisenberger (2002) found that perceived procedural justice has a strong positive relationship with perceptions of organizational support and assert that employees may perceive that justice is regulated by management. Thus, an important focus for new research is to understand the factors that contribute to maintaining positive organizational behaviors and attitudes such as procedural justice.

In the procedural justice literature, Ambrose and Schminke (2003) propose that organizations may enhance employees' procedural justice perceptions by drafting formal policies that provide details of how outcomes are determined (Rhoades and Eisenberger, 2002), as this offers employees some control over decision-making processes, and provides reassurance about the fairness of the resulting outcomes (Thibaut and Walker, 1975). Coyle-Shapiro and Conway (2005) posit that the tenet of psychological contract theory in the employee-employer relationship is a mutual exchange of obligations, along with a perception of the level to which those obligations are fulfilled. According to the authors (p. 775) the exchange process includes “any item that might be exchanged between the organization and the employee (e.g., pay, training, support, in exchange for loyalty, performance, flexibility).” The literature shows that the fulfillment of a psychological contract is positively related to satisfaction with the organization, jobs and leaders; to commitment to the organization; to trust in the organization; and to OCB, and is negatively related to turnover intentions (Coyle-Shapiro and Conway, 2005). Consistency between what is promised and provided by the organization establishes the basis of the reciprocal exchange (Pignata, 2011). Whilst perceived fairness is crucial for the relational psychological contract to continue (Rousseau and Parks, 1992), any violation of the contract and perception of unfulfilled obligations raises issues of procedural justice, and reduces the likelihood of employees engaging in civic virtue behaviors (see Robinson and Morrison, 1995). For example, assessments of organizational procedures, in terms of procedural justice, can influence an employee's trust (Folger and Konovsky, 1989; Saunders and Thornhill, 2003). In this way, perceptions of procedural justice can impact on the key determinants of trust: Integrity, ability, and benevolence. Thus, a perpetual sequence may exist amongst these constructs, where fair and beneficial organizational procedures encourage trust from employees, which then promotes pro-organizational behaviors that assist the organization to perform and achieve its goals. Some studies support this sequence as they show that perceptions of procedural justice promote trust, and that trust promotes affective organizational commitment (Bijlsma and Koopman, 2003).

### Justice and reciprocity

The tenet of Blau's (1964) social exchange theory is that people seek to reciprocate to those who benefit them. This state of reciprocal interdependence generates perceived obligations (Saks, 2006) as the voluntary action of providing something of value to another in a social exchange creates a perceived obligation on the other party to reciprocate. In the work setting, when employees see that fair procedures are implemented, they may feel valued by their organization and as a result, respond with increased feelings of commitment to the organization (Pignata et al., 2016). By contrast, perceptions of unfair procedures have been associated with decreased OCB and increased turnover intentions (Colquitt et al., 2001). Furthermore, Elovainio et al. (2001) assert that employees' perceptions of organizational injustice are associated with psychological strain at work. Siegrist's (1996) Effort-Reward Imbalance work stress model is based on the relationship between effort made at work and the reward obtained, and proposes that work strain is a consequence of an imbalance between the effort expended and the rewards gained. Effort is defined as the job demands and obligations that are imposed on the employee, with the premise of the model being that effort at work is reciprocated by adequate reward in the form of socio-emotional response (approval, respect, recognition, support), status, or money, as part of a social contract. A balance is achieved when the benefits received are what should be expected given the contribution at work. Work situations demanding high effort and offering little gain may have adverse effects on physical and emotional health due to the effort-reward imbalance (Marmot et al., 2002). For example, research on job satisfaction in Australian universities by Bentley et al. (2013b) highlighted key aspects of job dissatisfaction in the sector.

In the context of Australian universities, the negative impact of economic and management pressures on the wellbeing of university staff has been documented by Winefield et al. (2008) with a qualitative focus group study of 15 universities showing that university staff experienced high levels of stress, reporting insufficient funding and resources, fewer opportunities for career development, and reduced recognition and reward practices (Gillespie et al., 2001). According to Kinman and Jones (2008, p. 247), “…interventions restoring the balance between efforts expended and rewards received—thus improving employees' sense of fairness and reciprocity. In the university sector, such balance could be achieved by reducing extrinsic efforts and/or enhancing rewards such as esteem, promotion prospects, and job security,” or both. Given that procedural justice perceptions can shape the employee-employer relationship, it is important to focus specifically on employees' perceptions of procedural justice in universities to investigate what types of workplace, demographic or individual characteristics predict procedural justice, so that intervention strategies can address the issues of control and uncertainty in employees' perceptions of procedural justice (Thibaut and Walker, 1975).

The importance of examining ethical issues in organizational interventions is noted by Dewe (1994, p. 23) who stated that the development and evaluation of such interventions “appears to be hampered by issues of power, control and ethics.” Ethics is defined as “a system of moral principles, by which human actions and proposals may be judged good or bad, or right or wrong” (Delbridge, 2001, p. 378). Hence, ethics involves the application of certain principles to decide the right thing to do in the situation. Some of the key values in contemporary approaches to ethics are autonomy, responsibility, care, and justice (May et al., 1998). May and colleagues state that autonomy refers to individuals being true to their principles and acting in the way that they have chosen. Responsibility includes both a personal and social orientation as it concerns accountability for the consequences that individuals have explicitly and directly caused, and includes both the actions that they have explicitly performed and things they have failed to do. Care is oriented to those who cannot protect themselves, or to those individuals with whom they have interpersonal relationships. Like responsibility, justice has a personal and social orientation as it refers to giving each individual their due on the basis of that individual's legitimate rights. Those rights include contractual rights between two equal parties, and the fair distribution of goods and services in a society.

### Justice and psychological contracts

The literature highlights: The positive relationships between fulfilled psychological contracts and satisfaction with the organization, jobs and leaders; the positive work-related outcomes of organizational commitment, trust, and OCB; and the negative relationship with turnover intentions (Masterson et al., 2000; Coyle-Shapiro and Conway, 2005). Thus, psychological contracts are clearly of importance to the employee-employer relationship. A meta-analysis by Zhao et al. (2007) showed that psychological contract breach impacts affective reactions, which in turn, affect important work behaviors and attitudes that result in employees being less likely to maintain their commitment to the organization. This leads into the deontological concerns of procedural justice that involve treating employees with respect and fairness.

Deontological theories view duty as the primary morally relevant feature, rather than consequences. Kant (cited in Cohen, 2004, p. 41) argued that “morality is a matter of doing one's duty, regardless of consequences, and that duty itself is determined not by reference to consequences but by reference to consistency and the requirements of rationality.” For Kant, the pure moral law was related to duty and was independent of feelings and inclinations (Singer, 1994). An example of a deontological principle is the rule to treat others as we would like to be treated when it is used as a gauge for correct conduct that does not make reference to consequences (Cohen, 2004). Psychological contracts are linked to deontological concerns such as honesty and loyalty in that it is an employee's duty to carry out their work diligently, and it is an employer's duty to treat employees with respect. Fairness is another fundamental deontological principle, and so such ethical principles are tied into perceived procedural justice as that is the degree to which individuals perceive decisions to have been made according to fair procedures (Folger and Greenberg, 1985).

According to Brown et al. (2010, pp. 1589–90) procedurally just decisions are made with accuracy and without bias, and “Greenberg's (1994) conceptualization of procedural justice emphasized consistency, soliciting input, and the opportunity for two-way communication during implementation.” Leventhal's (1980) seminal work established six principles of procedural justice: (1) consistency in applying just policies to all employees over a period of time; (2) no bias from those making the decisions; (3) ensuring the accuracy and completeness of information used to make the decision; (4) providing the opportunity to appeal outcomes; (5) the opportunity for those affected to voice their views regarding the outcome; and (6) upholding the ethical standards of those affected by the decision. For example, in deciding who should receive a promotion, a failure to apply the promotion policy correctly or to be biased in the decision may evoke procedural unfairness concerns in the employee(s) affected by the decision. Research on justice, trust, and work stress in the Australian university context has often occurred during difficult periods of enterprise bargaining when academic staff, in particular, were sensitive to justice issues regarding their pay, working conditions, and their opportunities for promotion, so it is not surprising that procedural justice was identified as a highly salient form of justice for academic staff (Gillespie et al., 2001). It is proposed that on the basis of social-exchange theory we can learn about employees' perceptions of justice by investigating the factors that may lead to their judgments of procedures and policies within highly formalized university settings. To this end, the present study examines the predictors of perceived procedural justice among a sample of university employees.

### Job demands and resources

**Figure 1:**
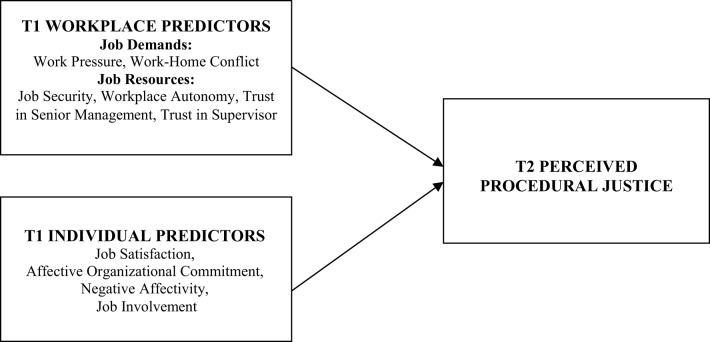
**Conceptual model of the hypothesized Time 1 predictors of Time 2 perceived procedural justice (adapted from Winefield et al., 2008)**.

In order to address a need for a theoretically based organizing framework, the conceptual model for the study (see Figure 1) integrates the reciprocal relationships in social exchange theory with the dual factor Job Demand-Resources (JD-R) model of workplace stress and engagement (Demerouti et al., 2001; Bakker and Demerouti, 2007). The JD-R framework has been employed in the present study as it comprises the two classes of job demands and job resources which are broad in scope as they encompass physical, social, psychological, and organizational dimensions of the job. Consistent with the model, the current study classifies job demands as parts of the job that require sustained effort or skills and entail physiological or psychological costs (Bakker and Demerouti, 2007). By contrast, it categorizes job resources as parts of the job that reduce demands, assist in attaining goals, and enhance growth and development which are crucial for employee wellbeing (Demerouti et al., 2001; Schaufeli and Bakker, 2004). Based on the work stress literature, predictors of procedural justice can include work pressure and work-home conflict (job demands). Work pressure is the sense of having too much work to do in the time available, which is often treated as an indicator of job demands (Demerouti et al., 2004). Work-home conflict refers to the processes by which an individual's behavior at home is negatively influenced by the demands of their work role. Work-home conflict is a key source of occupational stress (DeFrank and Ivancevich, 1998) and a predictor of exhaustion, health complaints and depressive affect (Geurts et al., 2003).

Conversely, job resources comprise perceptions of job security, job autonomy, trust in senior management, and trust in supervisor which are particularly pertinent to perceptions of procedural justice. Job security refers to expectations about job continuity, whereas job insecurity indicates negative expectations about job continuity, and in the university setting has been shown to predict both psychological strain and lower job satisfaction for academic and non-academic staff (Winefield et al., 2002). Job autonomy is the degree to which employees can influence the pacing and timing of their work (Bakker and Demerouti, 2007), and, along with procedural justice, has been shown to influence psychological strain and commitment to the organization in academics (Boyd et al., 2011). Mayer et al. (1995, p. 712) define trust as “the willingness of a party to be vulnerable to the actions of another party based on the expectation that the other will perform a particular action important to the trustor, irrespective of the ability to monitor or control that other party.” Trust in supervisor (in the university context typically a Head of Department) and trust in senior management refer to the extent to which supervisors or senior management are perceived by their subordinates to act with integrity, competence, and benevolence toward them. In their longitudinal study across three time-points, Holtz and Harold (2009, p. 1195) examined overall organizational and overall supervisory justice perceptions of 213 individuals, and found that “employees with higher levels of trust had more stable overall justice perceptions.” They also found that overall justice perceptions changed over time as shown by within-person variance in organizational and supervisory justice.

### Predictors

In the present study, along with job demands and job resources, the attitudinal variables of job satisfaction and organizational commitment were included as predictors of procedural justice. Job satisfaction refers to the broad positive emotional reactions and attitudes individuals have toward their jobs (Locke, 1969). In their research on job satisfaction around the academic world, Bentley et al. (2013a) showed that academics are committed to their work and find it deeply satisfying. Other studies within university settings have found that academic staff reported intrinsic factors such as student interaction, relationships with fellow colleagues, the prestige of academic positions, autonomy and job variety, as their areas of greatest satisfaction (Kinman, 2001; Winefield et al., 2002). As the main predictors of job satisfaction include trust in management, autonomy, and procedural justice, we predict an association between perceptions of procedural justice and the job resources of autonomy, trust in supervisor, and trust in senior management.

Another key attitude is affective organizational commitment which refers to “the strength of an individual's identification with and involvement in a particular organization” (Porter et al., 1974, p. 604). As commitment develops from employees' work experiences and can be enhanced by positive experiences (Arnold et al., 1995), it can also be expected to predict procedural justice. The Time 1 (T1) level of procedural justice was included as an additional predictor to ensure that tests of the relationships between the hypothesized predictors and outcomes are rigorous.

The conceptual model for the study also contained the individual characteristics of negative affectivity and job involvement as the literature demonstrates their relationship to occupational stress and wellbeing (see Winefield et al., 2002). Negative affectivity and job involvement have been included in the analyses as possible predictors of procedural justice as they have been shown to exert an impact on strain, burnout, job satisfaction and organizational commitment (Landsbergis et al., 1992). Negative affectivity, refers to “the disposition to experience aversive emotional states” (Watson and Clark, 1984, p. 465), and as a stable personality characteristic may influence work attitudes over time. As employees with high levels of negative affectivity may be more likely to confront their supervisor due to their need to control the outcomes they experience and/or reduce their feelings of uncertainty in achieving fair and appropriate procedural outcomes (e.g., salary increases and promotional opportunities), it is predicted that there will be a negative association between negative affectivity and procedural justice perceptions. Furthermore, job involvement is a function of both individual differences and job characteristics and is the extent to which employees prioritize and become involved in their work (Lodahl and Kejner, 1965). Hallberg and Schaufeli (2006) showed that job involvement is an aspect of work attachment that relates to an intrinsic need for performance. As research in an Irish academic context by Kanungo (1982) found that Irish academics report high levels of job involvement and that their work relates to their core identity, it can be speculated that this focus on their work role and self-identity may translate to them caring for fair procedures and thus having positive perceptions of procedural justice within their work setting. Indeed, it has recently been suggested by Ruokolainen et al. (2016) that with regard to psychological contracts, and perhaps due to their higher levels of education, university employees expect more of themselves and their employer than those who work in less demanding work settings.

Due to the aforementioned justice research, and as the tenet of the JD-R model is that high job demands are associated with negative individual and workplace outcomes, and that abundant job resources are associated with achieving beneficial outcomes for individuals and workplaces, the present study incorporated the abovementioned variables in a two-wave longitudinal design to examine the effects of perceived procedural justice over time in order to investigate the following hypotheses:

*Hypothesis 1*: Job demands (work pressure, work-home conflict) at T1 will be negatively associated with perceptions of procedural justice at T2.
*Hypothesis 2*: Job resources (job security, autonomy, trust in supervisor, trust in senior management) at T1 will be positively associated with perceptions of procedural justice at T2.
*Hypothesis 3*: Work attitudes (job satisfaction and affective organizational commitment) at T1 will be positively associated with perceptions of procedural justice at T2.
*Hypothesis 4*: Negative affectivity at T1 will be negatively associated with perceptions of procedural justice at T2.
*Hypothesis 5*: Job involvement at T1 will be positively associated with perceptions of procedural justice at T2.

In Australia, academic work includes training professionals, conducting scholarly and applied research, building international networks, collaborating with business, mentoring individuals, and contributing to broader economic development (Coates and Goedegebuure, 2010). Thus, due to their different functional roles, and as prior research (see Winefield et al., 2008) indicated that academic and non-academic (professional) staff experience working conditions somewhat differently, with academic staff reporting more adverse work experiences than non-academic staff, we will explore the possibility that the predictors of perceived procedural justice will differ for academic (faculty) and non-academic staff.

## Materials and methods

### Participants and procedure

A total sample of 4969 participants responded to both the 2000 (T1) and 2003/4 (T2) surveys at a representative sample of 13 Australian public universities (see Winefield et al., 2008 for more details and a broad analysis of the results of the survey). A three-year interval between the longitudinal surveys for 12 of the universities, and a four-year interval for one university was chosen in order to provide university management with adequate time to implement the researchers' recommendations for reducing occupational stress. Of the 4969 participants, this paper reports on the responses from the 945 matched tenured and contract staff (casual or hourly paid employees did not participate in the study) who participated in both waves of the survey and provided responses to the measure of perceived procedural justice. The mean age of the sample was 46.5 years with a standard deviation of 9.3 years. The sample comprised 345 men (37%) and 600 women (63%), and 399 (42%) academic (faculty) staff and 546 (58%) non-academic (professional) staff which is typical of the national Australian profile of academic and non-academic staff (Department of Education Science and Training, 2003). The longitudinal study received ethics approval from the University of South Australia's Human Research and Ethics Committee. As participation in both surveys was anonymous, code identifiers were used to match data across surveys.

### Measures

Both the T1 and T2 questionnaires sought demographic information (age, university), and the following attitude and individual difference measures in the survey. The means shown for job satisfaction and negative affectivity are based on the total scores, whereas the measures of work pressure, work-home conflict, job security, autonomy, affective organizational commitment, trust in senior management, trust in supervisor, job involvement, and perceived procedural justice are based on the item means. The items for the procedural justice measure were developed from focus group discussions (see Gillespie et al., 2001), and the findings of the focus group study were used to inform the design of the questionnaire. The measures used in the survey were taken from well validated scales that have been published in the literature however, some measures were modified to ensure that the items were sensitive and relevant to the university context. For example, with regard to the job security measure, we retained three items: (1) Lose your job and be laid off; (2) Find your department or division's future uncertain; (3) Lose your job by being pressured to accept early retirement, as the other items were not judged to be as relevant to staff who were, by definition, in relatively secure long-term employment. The same criterion was used for other survey measures.

The measures of direct relevance had internal reliabilities between 67 and 96 (Cronbach's alpha coefficients) indicating acceptable reliability (see Table 1). It should be noted that work pressure was strongly associated with work-home conflict (*r* = 0.65) but none of the correlations suggest that any of the self-report measures were assessing the same constructs (see DeVellis, 1991; Nunnally and Bernstein, 1994).

**Table 1:** **Descriptive statistics and correlations for demographic, individual characteristics, and workplace variables for all study participants**.

** Variable**	***Mean*^a^**	***SD***	**α**	**1**	**2**	**3**	**4**	**5**	**6**	**7**	**8**	**9**	**10**	**11**	**12**	**13**	**14**	**15**
1. Age	46.52	9.28	—															
2. Gender	0.63	0.48	—	−0.14^**^														
3. Tenure	12.10	7.34	—	0.46^**^	−0.09^*^													
4. Staff group	1.58	0.49	—	−0.20^**^	0.20^**^	−0.07^*^												
5. T1 Work pressure	3.22	0.63	0.78	0.17^**^	−0.06	0.15^**^	−0.33^**^											
6. T1 Work-home conflict	3.30	1.03	0.86	0.12^**^	−0.07^*^	0.10^**^	−0.39^**^	0.65^**^										
7. T1 Job security	2.59	0.92	0.68	−0.24^**^	0.04	−0.20^**^	0.04	−0.09^**^	−0.15^**^									
8. T1 Autonomy	3.07	0.54	0.70	−0.12^**^	0.06	−0.15^**^	−0.03	−0.09^**^	−0.14^**^	0.26^**^								
9. T1 Job satisfaction	66.43	13.22	0.87	−0.06	0.15^**^	−0.13^**^	0.18^**^	−0.28^**^	−0.41^**^	0.44^**^	0.57^**^							
10. T1 Organizational commitment	3.49	0.69	0.80	−0.01	0.08^*^	−0.09^**^	0.17^**^	−0.13^**^	−0.16^**^	0.20^**^	0.32^**^	0.49^**^						
11. T1 Trust in senior management	2.55	0.86	0.95	−0.08^*^	0.04	−0.18^**^	0.19^**^	−0.28^**^	−0.30^**^	0.26^**^	0.37^**^	0.55^**^	0.44^**^					
12. T1 Trust in supervisor	3.36	1.01	0.96	−0.04	−0.02	−0.09^**^	−0.02	−0.03	−0.13^**^	0.25^**^	0.41^**^	0.47^**^	0.18^**^	0.21^**^				
13. T1 Procedural justice	3.06	0.66	0.72	0.01	0.03	−0.06	−0.01	−0.06	−0.15^**^	0.26^**^	0.40^**^	0.54^**^	0.30^**^	0.41^**^	0.43^**^			
14. T1 Negative affectivity	19.33	7.82	0.88	−0.16^**^	0.06	0.01	0.00	0.07^*^	0.24^**^	−0.21^**^	−0.15^**^	−0.27^**^	−0.18^**^	−0.13^**^	−0.09^**^	−0.14^**^		
15. T1 Job involvement	2.86	0.67	0.67	0.21^**^	−0.11^*^	0.14^**^	−0.27^**^	0.22^**^	0.36^**^	−0.02	0.08^*^	0.05	0.22^**^	0.02	0.05	0.10^**^	0.08^*^	
16. T2 Procedural justice	3.08	0.81	0.80	−0.01	0.00	0.03	0.05	−0.01	−0.12^**^	0.21^**^	0.24^**^	0.42^**^	0.22^**^	0.32^**^	0.28^**^	0.52^**^	−0.12^**^	0.08^*^

*N = 728—945*.

a *For Job Satisfaction and Negative Affectivity, item scores were summed. Item scores were averaged for all other measures*.

* *p < 0.05*,

** *p < 0.01*.

#### Work pressure

Three items from Beehr et al.'s (1976) Work Pressure scale assessed work overload. An example item is “I don't have time to finish my job.” Questions were rated on a 4-point scale (1 = *definitely false*, 4 = *definitely true*).

#### Work-home conflict

Three items from Frone and Yardley's (1996) scale measured work-home conflict with an example item being, “My family dislikes how often I am preoccupied with my work while I am at home.” Each item was rated on a 5-point scale (1 = *never*, 5 = *very frequently*).

#### Job security

Three items drawn from Ashford et al.'s (1989) measure of job insecurity asked staff to rate the likelihood (1 = *very unlikely*, 5 = *likely*) of items such as losing their job and being laid off; finding your department/division's future uncertain; being pressured to accept early retirement. The items were reverse scored to be consistent with the other job resource measures so that high scores meant high job security.

#### Workplace autonomy

A 9-item measure, drawn from the Moos Work Environment Scale autonomy sub-scale (Moos and Insel, 1974), was used. An example item is “Staff are encouraged to make their own decisions,” and responses are rated on a 5-point scale (1 = *strongly disagree*, 5 = *strongly agree*).

#### Job satisfaction

The 15–item scale developed by Warr et al. (1979) assessed job satisfaction (e.g., “How satisfied or dissatisfied do you feel with the amount of variety in your job?”), and each item was scored on a 7–point Likert scale (1 = *extremely dissatisfied*; 7 = *extremely satisfied*).

#### Affective organizational commitment

Five items from Porter et al.'s (1974) scale measured affective organizational commitment with each item scored on a 5-point scale (1 = *strongly disagree*; 5 = *strongly agree*). An example item is “I really care about the future of this university.”

#### Trust in senior management

An 8–item scale developed from Mayer and Davis (1999) and Butler (1991) measured trust in senior management (e.g., “Senior Management of my University treat staff fairly”). Each item was scored on a 5-point scale (1 = *strongly disagree*; 5 = *strongly agree*).

#### Trust in supervisor

An 8–item scale adapted from Mayer and Davis (1999) and Butler (1991) measured trust in supervisor by assessing employees' perceptions of the level of integrity, competence, and concern for staff shown by their supervisor, school, or unit.

#### Procedural justice perceptions

A 4–item scale developed by Gillespie et al. (2001) measured perceptions of procedural justice (e.g., “Staff performance is fairly appraised”) on a 5-point scale (1 = *strongly disagree*; 5 = *strongly agree*).

#### Negative affectivity

The 12–item measure of Neuroticism from the NEO-Five Factory Inventory (NEO–FFI: Costa and McCrae, 1992) was used to measure negative affectivity, which assesses an individual's disposition to experience anxiety, depression and vulnerability. Each item was rated on a 5-point scale (0 = *strongly disagree*, 4 = *strongly agree*, with reverse scoring for positively phrased items).

#### Job involvement

Four items from the scale developed by Lodahl and Kejner (1965) measured the extent to which staff are involved in their work. An example item is “The most important things that happen to me involve my work.” Each item was rated on a 5-point scale (1 = *strongly disagree*, 5 = *strongly agree*).

#### Control variables

To reduce the possibility of spurious relationships with demographic characteristics, the control variables of age, gender (1 = *male*, 2 = *female*), length of organizational tenure, and staff group (1 = *academic*, 2 = *non-academic*) were entered in all the equations. The T1 level of procedural justice was also entered into each equation.

### Analyses

Analyses were performed using Predictive Analytics Software (PASW) 18.0. Preliminary checks ensured that there was no violation of the assumptions of multicollinearity, normality, linearity, and homoscedasticity. As prior levels of procedural justice at T1 were included as additional predictors in the analysis, tests of the aforementioned relationships are more rigorous as they show that the predictors account for changes in the levels of the dependent variables (Zapf et al., 1996).

## Results

The means, standard deviations, internal reliability coefficients and bivariate correlations for the variables are displayed in Table 1.

The descriptive statistics for the variables for both academic and non-academic staff are displayed in Table 2.

**Table 2:** **Mean (SD) age, gender, and all measures for academic and non-academic (professional) staff categories**.

	**Academic (*n* = 249)**		**Non-academic (*n* = 324)**	
	***M***	***SD***	***M***	***SD***
Age	48.34	7.96	44.94	9.26
Gender	0.51	0.50	0.69	0.46
Length of tenure	13.09	7.59	11.90	7.07
Staff group	1.02	0.15	1.98	0.15
T1 Work pressure	3.46	0.56	3.06	0.61
T1 Work-home conflict	3.82	0.98	2.93	0.91
T1 Job security	2.69	0.94	2.60	0.88
T1 Autonomy	3.04	0.53	3.06	0.55
T1 Job satisfaction	62.71	13.73	67.89	13.00
T1 Organizational commitment	3.36	0.74	3.55	0.63
T1 Trust in senior management	2.32	0.90	2.65	0.78
T1 Trust in supervisor	3.35	1.07	3.28	0.99
T1 Procedural justice	3.06	0.68	3.04	0.70
T2 Procedural justice	3.06	0.81	3.16	0.80
T1 Negative affectivity	19.67	8.19	19.10	7.64
T1 Job involvement	3.06	0.67	2.71	0.64

*Coding: Gender (Male = 1, Female = 2), Staff Group (Academic = 1, Non-academic = 2)*.

The hierarchical regression analyses investigating the predictors of T2 procedural justice for both staff categories are displayed in Table 3.

**Table 3:** **Multiple regression analyses of all predictors of T2 procedural justice for all study participants**.

**Predictors**	**Step 1**					**Step 2**					**Step 3**				
	***B***	***SE B***	**β**	***t***		***B***	***SE B***	**β**	***t***		***B***	***SE B***	**β**	***t***	
Age	−0.01	0.02	−0.04	−0.77		−0.03	0.02	−0.07	−1.63		−0.03	0.02	−0.08	−1.84	
Gender	−0.11	0.28	−0.02	−0.36		−0.25	0.24	−0.04	−1.03		−0.21	0.25	−0.03	−0.86	
Tenure	0.02	0.02	0.05	1.06		0.04	0.02	0.09	2.28^*^		0.04	0.02	0.09	2.30^*^	
Staff group	0.35	0.28	0.05	1.27		0.31	0.26	0.05	1.19		0.34	0.27	0.05	1.28	
T1 Work pressure						0.50	0.24	0.10	2.07^*^		0.47	0.24	0.09	1.96^*^	
T1 Work-home Conflict						0.05	0.16	0.01	0.29		0.06	0.17	0.02	0.34	
T1 Job security						0.05	−0.15	0.01	0.35		0.03	−0.15	0.01	0.20	
T1 Autonomy						−0.48	0.27	−0.08	−1.78		−0.49	0.27	−0.08	−1.82	
T1 Job satisfaction						0.05	0.02	0.22	3.58^***^		0.05	0.02	0.22	3.50^**^	
T1 Org commitment						−0.15	0.20	−0.03	−0.75		−0.20	0.20	−0.04	−0.97	
T1 Trust in snr management						0.36	0.17	0.09	2.13^*^		0.36	0.17	0.09	2.14^*^	
T1 Trust in supervisor						0.12	0.14	0.04	0.92		0.13	0.14	0.04	0.96	
T1 Procedural justice						0.46	0.05	0.39	8.82^***^		0.46	0.05	0.39	8.79^***^	
T1 Negative affectivity											−0.02	0.02	−0.04	−1.01	
T1 Job involvement											0.10	0.20	.02	0.049	
*Adj. R*^2^					−0.00										
*F*					0.77										
Δ*R*^2^										0.31					
*F change*										28.98^***^					
Δ*R*^2^															0.00
*F change*															0.59

*N = 573.ΔR^2^ = change in R^2^*.

* *p ≤ 0.05*.

** *p ≤ 0.01*.

*** *p ≤ 0.001*.

As shown in Table 3, employee perceptions of procedural justice at T2 were predicted by workplace factors that predicted 31% of the variability in procedural justice. The individual characteristics did not account for the variance in justice. Excluding the effects of the T1 level of procedural justice, the strongest predictor of justice was job satisfaction which means that the more satisfied that employees are with their jobs, the higher their perceptions of procedural justice. University employees' trust in their senior management, and their length of organizational tenure predicted justice as longer tenured employees perceived higher levels of justice. Of particular note, and unexpectedly, work pressure also positively predicted procedural justice.

The hierarchical regression analyses investigating the predictors of T2 procedural justice for academic staff are displayed in Table 4.

**Table 4:** **Multiple regression analyses of all predictors of T2 procedural justice for the academic staff category**.

**Predictors**	**Step 1**					**Step 2**					**Step 3**				
	***B***	***SE B***	**β**	***t***		***B***	***SE B***	**β**	***t***		***B***	***SE B***	**β**	***t***	
Age	−0.03	0.03	−0.08	−1.01		−0.03	0.03	−0.06	−1.01		−0.06	0.03	−0.09	−1.32	
Gender	0.32	0.42	0.05	0.76		−0.12	0.36	−0.02	−0.34		0.01	0.37	0.00	0.03	
Tenure	0.02	0.03	0.06	0.72		0.04	0.03	0.09	1.31		0.04	0.03	0.09	1.40	
T1 Work pressure						0.85	0.39	0.14	2.18^*^		0.76	0.39	0.13	1.94	
T1 Work-home conflict						−0.28	0.23	−0.07	−0.99		−0.30	0.25	−0.09	−1.16	
T1 Job security						0.03	−0.21	0.01	0.15		0.01	−0.22	0.00	0.03	
T1 Autonomy						−0.14	0.41	−0.02	−0.35		−0.14	0.41	−0.02	−0.33	
T1 Job satisfaction						0.05	0.02	0.22	2.37^*^		0.05	0.02	0.20	2.10^*^	
T1 Organizational commitment						0.03	0.27	0.01	0.10		−0.10	0.28	−0.02	−0.36	
T1 Trust in senior management						−0.08	0.23	−0.02	−0.35		−0.05	0.23	−0.01	−0.21	
T1 Trust in supervisor						−0.02	0.19	−0.01	−0.10		0.02	0.20	0.01	0.12	
T1 Procedural justice						0.55	0.08	0.45	6.51^***^		0.54	0.08	0.45	6.44^**^	
T1 Negative affectivity											−0.02	0.02	−0.06	−0.98	
T1 Job involvement											0.44	0.31	0.09	1.45	
*Adj. R*^2^					−0.01										
*F*					0.54										
Δ*R*^2^										0.36					
*F change*										14.92^***^					
Δ*R*^2^															0.01
*F change*															1.43

*N = 249. ΔR^2^ = change in R^2^*.

* *p ≤ 0.05*.

** *p ≤ 0.01*.

*** *p ≤ 0.001*.

For academic staff, Table 4 shows that workplace factors predicted 36% of the variance in justice. After controlling for the T1 level of justice at Step 2, job satisfaction and work pressure were the strongest predictors of justice perceptions, however after the inclusion of the individual difference factors of negative affectivity and job involvement, the effects of work pressure did not endure and only job satisfaction remained a predictor of justice.

The hierarchical regression analyses investigating the predictors of perceived procedural justice for non-academic staff are displayed in Table 5.

**Table 5:** **Multiple regression analyses of all predictors of T2 procedural justice for the non-academic (professional) staff category**.

**Predictors**	**Step 1**					**Step 2**					**Step 3**				
	***B***	***SE B***	**β**	***t***		***B***	***SE B***	**β**	***t***		***B***	***SE B***	**β**	***t***	
Age	−0.01	0.02	−0.02	−0.36		−0.03	0.02	−0.08	−1.37		−0.03	0.02	−0.08	−1.32	
Gender	−0.48	0.39	−0.07	−1.23		−0.48	0.34	−0.07	−1.41		−0.49	0.35	−0.07	−1.40	
Tenure	0.03	0.03	0.07	1.08		0.05	0.02	0.11	2.16^*^		0.05	0.02	0.11	2.20^*^	
T1 Work pressure						0.36	0.31	0.07	1.17		0.33	0.31	0.06	1.05	
T1 Work-home conflict						0.27	0.21	0.08	1.27		0.32	0.23	0.09	1.43	
T1 Job security						0.06	−0.21	0.02	0.30		0.06	−0.21	0.02	0.29	
T1 Autonomy						−0.67	0.37	−0.12	−1.9		−0.69	0.38	−0.12	−1.82	
T1 Job satisfaction						0.06	0.02	0.23	2.77^**^		0.06	0.02	0.23	2.76^**^	
T1 Organizational commitment						−0.58	0.29	−0.12	−1.99^*^		−0.55	.31	−0.11	−1.78	
T1 Trust in senior management						0.89	0.25	0.22	3.58^***^		0.89	0.25	0.22	3.57^***^	
T1 Trust in supervisor						0.19	0.19	0.06	1.01		0.21	0.19	0.06	1.06	
T1 Procedural justice						0.39	0.07	0.34	5.85^***^		0.39	0.07	0.34	5.86^***^	
T1 Negative affectivity											−0.01	0.02	−0.02	−0.33	
T1 Job involvement											−0.16	0.27	−0.03	−0.61	
*Adj. R*^2^					−0.00										
*F*					0.88										
Δ*R*^2^										0.30					
*F change*										15.37^***^					
Δ*R*^2^															0.00
*F change*															0.27

*N = 324. ΔR^2^ = change in R^2^*.

* *p ≤ 0.05*.

** *p ≤ 0.01*.

*** *p ≤ 0.001*.

Table 5 shows that for non-academic staff, workplace factors predicted 30% of the variance in justice perceptions. Consistent with the results for academic staff, job satisfaction was the strongest predictor of higher perceptions of procedural justice. However, there were differences between the two staff groups as non-academic employees' level of trust in their senior management and their length of organizational tenure also predicted perceived procedural justice. Indeed, in none of the three hierarchical regression analyses was negative affectivity nor job involvement related to employee perceptions of procedural justice. However, an unexpected finding was the negative relationship between non-academic employees' levels of organizational commitment and their justice perceptions. Whilst the effect of lower levels of commitment on justice did not endure when the individual characteristics were added to the analyses at Step 2, this unexpected result warrants further investigation.

## Discussion

This study employed workplace and individual difference characteristics through the theoretical frameworks of social exchange and the JD-R model to examine the factors that predicted employees' perceptions of procedural justice in the university setting. With regard to hypothesis 1, and contrary to expectations, the job demand of work pressure positively predicted procedural justice for the “all staff” category which suggests that university employees who may feel under work or time pressure have higher perceptions of the fairness and justice of procedures than those who feel less pressured by work. While this result was not evident in the separate analyses for each of the two staff categories, it is of interest and warrants further investigation particularly in terms of the negative impact of job demands on employee health and wellbeing as shown by the JD-R research literature, and due to the negative effect of perceptions of unfair procedures in terms of decreased OCB, increased turnover intentions (Colquitt et al., 2001), and increased levels of psychological strain at work (Elovainio et al., 2001).

Hypothesis 2 was partially supported as employees' trust in senior management was positively associated with perceptions of justice which supports existing research (Folger and Konovsky, 1989; Saunders and Thornhill, 2003; Holtz and Harold, 2009). In addition, the demographic factor of an employees' length of organizational tenure predicted justice. The reason for this may be that as trust in senior management and perceptions of justice develop over time through the repeated interactions between employee-organization, it is likely that employees who trust senior management and perceive things to be done fairly are more likely to stay longer in the job. The results reveal the importance of workplace factors in enhancing fair procedures to encourage reciprocity from university employees. For the “all staff” category, and in partial support of hypothesis 3, job satisfaction was a dominant and enduring predictor of perceived procedural justice.

The two distinct staff categories (academic, non-academic) were analyzed separately in order to explore any occupation specific effects due to their different functional roles. There were differences in the predictors of procedural justice for academic and non-academic staff, as workplace factors for academics predicted 36% of the variance in justice perceptions, and job satisfaction and work pressure were the strongest predictors of perceived justice, however after the inclusion of the individual difference factors of negative affectivity and job involvement, only job satisfaction predicted perceived justice, partially supporting hypothesis 3. As there have been long-term concerns about the low levels of job satisfaction in Australian academics (Bentley et al., 2013b), this study identifies the important link between job satisfaction and perceptions of justice. For non-academic staff, workplace factors predicted 30% of the variance in justice perceptions, and consistent with the results for academic staff, and again in partial support of hypothesis 3, job satisfaction was the strongest predictor of higher perceptions of procedural justice. Nevertheless, there were differences between the two staff groups as non-academic employees' trust in their senior management (partially supporting hypothesis 2) and their length of organizational tenure predicted perceived justice. This result is supported by the organizational justice and trust literature and suggests that longer tenure employees have come to understand and appreciate the values represented by the organization (O'Reilly and Chatman, 1986). The findings are also consistent with prior research that academic employees report more adverse work experiences than non-academic employees (Winefield and Jarrett, 2001; Winefield et al., 2003, 2008).

With regard to hypotheses 4 and 5, it is of interest that there were no associations between perceptions of procedural justice and the individual characteristics of negative affectivity and job involvement. The result for job involvement is particularly surprising given the strong association between an academics' work and their self-identity and that van Prooijen et al. (2002) found that social status was an important factor in procedural fairness concerns. Future research is needed in this area and there have been calls by Zhao et al. (2007) for research to also examine individual personality variables which may be particularly relevant in employees' affective reactions to breaches.

### Implications

The strength of this study is its longitudinal design which provides results with practical implications for management. The results highlight the importance of workplace factors in enhancing the perceptions of fair procedures to encourage reciprocity from employees. Indeed, research has identified people's need to reduce uncertainty as the key component of why procedural justice is important to them (De Cremer and Blader, 2006). As procedural justice perceptions are also conceptually linked to the psychological contract between employees and employers, it is possible that employees' levels of job satisfaction and their perceptions of trust in senior management, relative to other work attitude outcomes, may be more effective for improving the broader working environment, and promoting staff wellbeing and morale. Given that prior psychological contract research has lacked “a theoretically based organizing framework” (Zhao et al., 2007, p. 649), the present study's integration of the JD-R theoretical framework demonstrates some progress in the area.

From an applied perspective, the importance of the workplace predictors identified in the study may assist university management to maintain or enhance current procedures and strategies. Organizations should look to ensure that processes are fair, transparent and just to increase justice perceptions. As noted in the literature (Bijlsma and Koopman, 2003), employee perceptions of trust in senior management and perceptions of procedural justice evolve over time through the repeated interactions between an employee and their employer, hence a strength of the study is the longitudinal approach that examines the effects of procedural justice perceptions across time.

The present study adds to research on applied business ethics as it focuses on the ethical aspects of procedural justice, psychological contracts, and the normative moral principles of autonomy, equality, and respect for individuals within employment relationships. Thibaut and Walker (1975, p. 212) posit that “the enactment of fair procedures is associated with the belief that one will be able to control one's own outcomes.” By applying ethical principles consistent with Leventhal's (1980) principles of procedural justice, strategies for enhancing employee perceptions of procedural justice and thereby providing control, should include involving employees in the redesign of existing procedures, greater transparency and employee voice and participation in their implementation, consistency in applying the decisions, and clearer communication of the benefits of the procedures to employees.

### Limitations

While the longitudinal design represents a methodological advantage, it is important to acknowledge that other uncontrolled characteristics of the sample may have influenced the results. The longitudinal analysis necessitated that participants remained employed at the same university for the period of the study, and that a sufficient number of employees were willing to respond to both waves of the data collection. It is possible that some participants, whose perceptions of justice may have declined significantly prior to the second wave, either left the university or failed to respond. Universities with high turnover, low response rates, or initially small staff numbers were thus probably under-represented in our sample. Though the study provides useful insights into the justice process, it has limitations. First, the data were collected by a self-report questionnaire that may be affected by social desirability. Second, the study was undertaken in the complex and hierarchical settings of universities with multiple faculties and tiers of management, and extensive numbers of employees so caution must be exercised in generalizing the findings to smaller and less structured organizations. Furthermore, and as asserted by Ruokolainen et al. (2016) universities are knowledge-based organizations in which employees may believe that it is their obligation to ensure their own wellbeing and to inform their employer about any concerns.

### Future research

Our findings provide an important insight into the specific workplace factors and processes that promote perceptions of justice in organizations. Given that Zhao et al. (2007) found that negative emotional reactions are a likely consequence of psychological contract breaches, future research is needed to investigate the role of procedural justice within organizations and, from an exchange perspective more broadly, how individual and workplace factors may function as resources to aid an organization to discharge its psychological contracts with employees by providing fair and just procedures. There is also a need to gain an important insight into the emotional reactions to procedural justice, specifically breaches of procedural justice.

In conclusion, social exchange theory assumes that relationships are based on reciprocity, whereby a positive behavior is expected to be met with a positive response of equal benefit, followed by a continual exchange and interdependence between the two parties. This study adds to research on applied business ethics as it focuses on the ethical aspects of procedural justice and highlights the importance of workplace factors in enhancing fair procedures in organizational policy to encourage reciprocity from employees and the positive consequences of increasing employees' performance. An organization's employees are its most valuable resource and the effectiveness of organizations depends on healthy and engaged employees (Kinman, 1998). Hence, it is important that organizations focus their attention on implementing fair policies to enhance employees' prosocial and extra-role behaviors. Therefore, universities, as well as other organizations, need to continue to address the perceptions and experiences of staff in order to achieve long-term improvements in staff attitudes, and the corresponding benefits to organizational health and performance.

## Author contributions

All four authors, SP, AW, CP, and CB contributed to the formulation and writing of this paper. SP also undertook the analyses.

### Conflict of interest statement

The authors declare that the research was conducted in the absence of any commercial or financial relationships that could be construed as a potential conflict of interest.
